# First report of *blaOXA-48* producing *Klebsiella pneumoniae* isolates from wastewater in Morocco

**DOI:** 10.4102/jphia.v15i1.598

**Published:** 2024-08-08

**Authors:** Amine Aiddi, Ilham Zerdani, Aboubakr Khazaz, Ihssane Benzaarate, Hafsa Mguild, Fatna Bourjilat, Kaotar Nayme

**Affiliations:** 1 Microbiology Unit, Laboratory of Ecology and Environment, Department of Biology, Faculty of Sciences Ben M’sik, Hassan II University, Casablanca, Morocco; 2 Molecular Bacteriology Laboratory, Center for Serums and Vaccines (Institut Pasteur du Maroc), Casablanca, Morocco; 3 Microbiology and Antimicrobial Agents Research Team, LB2VE, Department of Biology, Faculty of Sciences, Chouaib Doukkali University, El Jadida, Morocco; 4 Research Laboratory of Microbiology, Infectious Diseases and Microbial Valorization, Mohammed VI University of Sciences and Health, Casablanca, Morocco; 5 Medical Bacteriology Laboratory, Center for Serums and Vaccines (Institut Pasteur du Maroc), Casablanca, Morocco

*Dear Editor,*

*Klebsiella pneumoniae* has emerged as an opportunistic pathogen responsible for a range of infections in both community and hospital settings. The spread of carbapenem-resistant *K. pneumoniae* (CRKP) strains, particularly those harbouring the *blaOXA-48* gene, poses a significant risk to public health and is particularly concerning for individuals with compromised immune systems or critical illnesses as it is associated with high mortality rates.^1^ The World Health Organization has classified CRKP alongside *Enterococcus faecium, Staphylococcus aureus, Acinetobacter baumannii, Pseudomonas aeruginosa* and *Enterobacter* spp., as a critical cluster of pathogens known as ESKAPE. This classification is because of their significant role in hospital-acquired infections and their high levels of antibiotic resistance, highlighting the pressing necessity for the development of novel antimicrobial agents to combat the escalating threat of antimicrobial resistance.^2^

The *blaOXA-48* gene encodes a carbapenemase enzyme that confers resistance to carbapenem antibiotics, which are often considered a last resort for treating severe bacterial infections.^3^ OXA-48 enzyme kinetics have shown high hydrolytic activity against penicillins and carbapenems, especially ertapenem, which is considered the most favourable substrate for this enzyme compared to imipenem and meropenem.^4^ Since the initial characterisation of the OXA-48 carbapenemase in a *K. pneumoniae* clinical isolate in Turkey in 2004,^5^ there has been a significant prevalence of bacterial strains that produce this enzyme in both nosocomial and community outbreaks across various regions of the world, particularly in the Mediterranean region and European countries.^6^ The rapid dissemination of *Enterobacteriaceae* strains that produce OXA-48-like enzymes in diverse ecosystems has recently emerged as a pressing concern. The number of reservoirs harbouring such organisms is on the rise not only within healthcare facilities but also within the community, among animals (e.g., livestock, companion animals and wildlife), and in the environment.^6^

## Methods

In order to gain insights into the occurrence of CRKP isolates in the environment, 10 wastewater samples were collected at six sampling sites in a sewage canal located in Casablanca City (Morocco) (coordinates: latitude: 33.519931 and longitude: −7.663718) in February 2024. Most of the sampling sites were impacted by pollution from agricultural, industrial and domestic origins.

A volume of 100 mL of wastewater was collected at each site under sterile conditions. The samples were immediately transported to the laboratory in an ice box for further analyses. Wastewater samples were processed for microbiological analysis following the TRIuMPH – SOP 2022 guidelines.^7^ Briefly, 500 μL of each sample was inoculated on brain heart infusion (BHI) and incubated at 37 °C for 24 h. The next day, one loopful (10 μL loop) from the BHI enrichment broth was inoculated on Brilliance UTI Clarity Agar (Oxoid), a chromogenic medium supplemented with ertapenem (0.5 μg/mL) and incubated at 37 °C for 24 h. Presumptive *K. pneumoniae* colonies were identified and confirmed using the VITEK 2 system (bioMerieux).

The antibiotic susceptibility patterns of the isolates were determined using the disc diffusion method on Mueller–Hinton agar (Bio-Rad) according to the recommendations of the European Committee (https://www.sfm-microbiologie.org/wp-content/uploads/2023/06/CASFM2023_V1.0.June_2023) The antibiotics disks (Oxoid) tested include temocillin (30 µg), ceftazidime (30 µg), piperacillin and tazobactam (30/6 µg), ceftazidime and avibactam (10/4 µg), cefepime (30 µg), aztreonam (30 µg), ertapenem (10 µg), imipenem (10 µg), meropenem (10 µg), ciprofloxacin (5 µg), gentamicin (10 µg), amikacin (30 µg), tigecycline (15 µg) and trimethoprim and sulfamethoxazole (125/2375 µg). Intrinsic resistance was not considered for susceptibility analysis (ampicillin and ticarcillin), as the focus was on identifying acquired resistance, which has significant clinical implications for treatment and infection control.^8^ Bacterial isolates resistant to three or more antimicrobial classes were catalogued as multidrug-resistant (MDR).^8^

To confirm the results obtained by the disc diffusion method, the determination of minimal inhibitory concentrations (MICs) was conducted using the Sensititre microdilution method (Sensititre™ FRAM2GN Thermo Fisher Diagnostics B.V., Scheepsbouwersweg 1 B, 1121 PC Landsmeer, the Netherlands). The interpretation of antimicrobial susceptibility results was based on the clinical breakpoints established by the European Committee on Antimicrobial Susceptibility Testing clinical breakpoints 2023 (https://www.sfm-microbiologie.org/wpcontent/uploads/2023/06/CASFM2023_V1.0.June_2023). KP ATCC 23357 was used as a wild-type susceptible control.

The carbapenemase activity among CRKP was detected using the Carba NP rapid diagnostic technique (Rapidec, Biomerieux, Marcy-l’Étoile, France), and the Oxa-48 production was confirmed using the Carba NG test (NG.BIOTECH Laboratories, Guipry, France).

**FIGURE 1:**
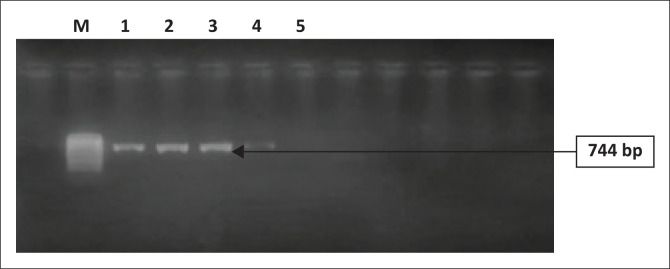
Polymerase chain reaction products of the amplification of *blaOxa-48* gene. Lane 1: 100 bp molecular weight marker; lanes 1 and 4: positive control; lanes 2 and 3: positive KP S8 and KP S19 isolates; lane 5: negative control. bp, base pairs.

Deoxyribonucleic acid (DNA) extraction was carried out from a bacterial culture in Luria-Bertani (LB) broth at 37 °C overnight using a genomic DNA extraction kit HigherPurity™ bacterial genomic DNA isolation kit (Canvax Biotech, S.L., Cordoba, Spain). All *K. pneumoniae* isolates were subjected to end-point polymerase chain reaction (PCR) to determine the presence of carbapenemase-encoding genes (*bla*KPC, *bla*NDM, *bla*VIM, *bla*IMP, *bla*OXA-48-like) as described previously.^9^ The amplicons were visualised on 1% agarose gels using a 1 kb Plus II DNA Ladder (TransGen Biotech, Haidian District, Beijing, China) to identify the PCR products (Figure 1).

## Results

A total of 10 *K. pneumoniae* isolates were obtained, among them 2 (20%) *K. pneumoniae* strains (KP S8, KP S19) were found to be OXA-48 producers. The antimicrobial susceptibility testing results revealed that among the studied isolates, KP S8 and KP S19 were resistant to various antibiotics including temocillin, ceftazidime, piperacillin-tazobactam, cefepime, aztreonam, tigecycline, trimethoprim-sulfamethoxazole and ceftolozane and tazobactam. Furthermore, these isolates were also resistant to ertapenem and had intermediate susceptibility to imipenem and meropenem. However, they remained susceptible to colistin, amikacin and ceftazidime and avibactam (Table 1). Both the Carba NG test and PCR confirmed the presence of bla*Oxa-48* in KP S8 and KP S19 (Figure 1).

**TABLE 1:** The distribution of the minimal inhibitory concentration values of 16 antibiotics tested against carbapenem-resistant *Klebsiella pneumoniae* strains harbouring *blaOXA-48-like*.

Strains code	MICs (mg/L)																Resistance gene detected
	TRM	TAZ	PTZ	CZA	FEP	AZT	ETP	IMI	MEM	CIP	GEN	AMI	TGC	COL	CLT	SXT	
KP S8	> 32	32	> 16	< 0.125	8	> 32	> 1	2	I	4	4	2	2	0.5	2	> 4	blaOXA-48-like
KP S19	> 32	32	> 16	< 0.125	8	> 32	> 1	2	2	0.25	8	4	4	0.5	4	> 4	blaOXA-48-like

TRM, temocillin; TAZ, ceftazidime; PTZ, piperacillin/tazobactam; CZA, ceftazidime/avibactam; FEP, cefepime; AZT, aztreonam; ETP, ertapenem; IMI, imipenem; MEM, meropenem; CIP, ciprofloxacin; GEN, gentamicin; AMI, amikacin; TGC, tigecycline; COL, colistin; CLT, ceftolozane/tazobactam; SXT, trimethoprim/sulfamethoxazole; MICs, minimal inhibitory concentrations.

## Conclusion

The detection of MDR *K. pneumoniae* strains harbouring the *blaOXA-48* gene in wastewater from Casablanca, Morocco, represents a significant public health concern. Our study is the first to report the presence of these strains in this region. This finding is consistent with previous studies that have documented the environmental presence of carbapenemase-producing *Enterobacteriaceae* (CPE). For instance, a study conducted in Algeria identified *blaOXA-48* producing *K. pneumoniae* in river water,^10^ suggesting a significant overlap between environmental and clinical reservoirs. Similarly, research from Spain reported the presence of *blaOXA-48* in wastewater treatment plants, emphasising the role of environmental pathways in the dissemination of antimicrobial resistance.^11^ Further genomic studies are needed to identify the sequence types of the isolates. Our findings emphasise the urgent need for comprehensive surveillance and targeted public health interventions to mitigate the spread of MDR organisms. Addressing this issue is crucial for protecting public health and combating the escalating threat of antimicrobial resistance.
